# Novel Surgical Approach for Limbal Dermoid Excision: Utilizing Bowman’s Membrane Lenticule and Autologous Limbal Stem Cell Transplantation for Enhanced Epithelial Healing and Visual Outcomes

**DOI:** 10.3390/vision9030056

**Published:** 2025-07-11

**Authors:** Dharamveer Singh Choudhary, Maya Hada, Kavita Ghanolia, Jeba Shaheen, Ajay Dhakad, Bhuvanesh Sukhlal Kalal

**Affiliations:** 1 Department of Ophthalmology, Swai Man Singh Medical College and Hospitals, Jaipur 302004, India; 2 Department of Biotechnology, Swai Man Singh Medical College and Hospitals, Jaipur 302004, India; 3 Department of Pharmacology and Nutritional Sciences, College of Medicine, University of Kentucky, Lexington, KY 40536, USA

**Keywords:** limbal dermoid, Bowman’s membrane, limbal stem cell transplantation, corneal reconstruction, AutoSLET

## Abstract

Limbal dermoids are congenital, benign, choristomatous growths affecting the corneal-limbal junction. Conventional excision techniques often result in persistent epithelial defects, corneal thinning, and vascularization due to sectoral limbal stem cell deficiency. This study investigated a novel surgical approach for limbal dermoid excision, utilizing Bowman’s membrane lenticule and autologous limbal stem cell transplantation, aimed at improving epithelial healing and visual outcomes. Thirty-four subjects (24 females, 10 males; mean age 8.33 ± 6.47 years) with limbal dermoids underwent the procedure. After dermoid excision, a Bowman’s membrane lenticule was placed over the defect and tucked 1 mm beneath the surrounding tissue. Sectoral limbal reconstruction was then performed using the AutoSLET technique. Pre- and postoperative assessments included visual acuity, corneal thickness, and epithelialization time. Statistical analysis employed paired *t*-tests. The mean epithelialization time was 3.36 ± 0.74 weeks, indicating rapid healing. Best-corrected visual acuity (BCVA) significantly improved from a preoperative mean of 0.136 ± 0.121 decimal units to a postoperative mean of 0.336 ± 0.214 decimal units (*p* < 0.001). Corneal thickness also demonstrated a significant increase, rising from a preoperative mean of 294 ± 49.68 microns to a postoperative mean of 484 ± 5.037 microns (*p* < 0.001). There is a transient edema below the Bowman lenticule observed in many cases, which resolves with deposition of granulation tissue. The findings suggest that the combined use of Bowman’s membrane lenticule and autologous limbal stem cell transplantation offers a promising surgical strategy for limbal dermoid excision. This technique promotes rapid epithelialization and leads to significant improvements in visual acuity and corneal thickness compared to conventional methods. The utilization of Bowman’s membrane as a natural basement membrane and the direct application of limbal stem cells facilitate enhanced epithelial healing and visual rehabilitation. While the study is limited by its small sample size, the results demonstrate the potential of this novel approach in managing limbal dermoids effectively.

## 1. Introduction

Corneal limbal dermoids, also known as epibulbar dermoids, are the most common congenital episcleral choristomas encountered in pediatric ophthalmology [1]. These lesions represent a developmental anomaly characterized by the presence of normally occurring tissues in an abnormal location on the globe [2]. They can manifest unilaterally or bilaterally, and may present as single or multiple lesions. These benign tumors arise from a combination of ectodermal and mesodermal tissues. Histologically, they are distinguished by a stratified squamous epithelium lining, often containing mature skin appendages such as hair follicles and sebaceous glands, and a lumen filled with keratin and hair. Dermoid cysts are typically congenital, presenting within the first year of life, and exhibit a slow, progressive growth pattern [3]. The pathogenesis involves an abnormal alteration in fetal development occurring around the 5th to 10th week of gestation, resulting in metaplastic transformation of the mesoblast situated between the rim of the optic nerve and the surface ectoderm. Epibulbar dermoids are classified into three grades based on their extent and depth of involvement: Grade I limbal dermoids are superficial and measure less than 5 mm; Grade II limbal dermoids cover more of the cornea and extend down to Descemet’s membrane; and Grade III limbal dermoids cover the entire cornea and extend through it to the anterior chamber, potentially reaching the pigmented epithelium of the iris [4]. Clinically, they present as pale, flesh-colored, pearly, dome-shaped, firm, deep-seated, subcutaneous nodules. These lesions are usually asymptomatic, non-pulsatile, and non-compressible. The presence of hair protruding from a punctum is considered pathognomonic for dermoid cysts [5]. Surgical intervention is indicated in cases where amblyopia (reduced visual acuity due to abnormal visual development) is unresponsive to conservative management, dellen formation (corneal thinning and dehydration), recurrent conjunctivitis, growth into the optical zone leading to visual impairment, inadequate lid closure causing exposure keratopathy, and for cosmetic reasons to improve the patient’s appearance and psychosocial well-being [6,7]. This study was designed to assess the healing process of the ocular surface following grade 1 dermoid excision, specifically evaluating the efficacy of a novel surgical technique involving the placement of a Bowman’s membrane lenticule and sectoral limbal reconstruction using autologous limbal stem cells [8]. The underlying hypothesis was that the Bowman’s membrane (BM) lenticule would provide a robust, natural, and innate basement membrane, facilitating epithelial migration, growth, stratification, stabilization, and maturation, thereby mitigating inflammatory insults to the epithelium and adjacent corneal tissue. Furthermore, the direct transplantation of limbal stem cells onto the new substrate was expected to accelerate epithelial recovery.

## 2. Materials and Methods

### 2.1. Study Design

This study was a hospital-based, non-randomized, prospective study, between January 2019 to July 2024 in the Department of Ophthalmology, Swai Man Singh Medical College and Hospitals, a tertiary care centre of North India. Prior to commencement, appropriate ethical clearance was obtained in accordance with the Declaration of Helsinki (Ref No.3968/MCEC/2018). All subjects were provided with detailed information regarding the surgical technique, its potential visual prognosis, associated risks, and benefits. Informed written consent was obtained from each participant.

### 2.2. Subjects Selection

Only subjects with Grade 1 limbal dermoids were included, as these cases presented with specific indications for surgery, such as significant corneal astigmatism, visual impairment, risk of amblyopia, or cosmetically concerning lesions prompting parental request. Primary indication was aesthetic appearance and higher grade of dermoids required larger procedures like DALK. Inclusion criteria encompassed subjects diagnosed with limbal dermoids who provided informed consent. Exclusion criteria included subjects who were bedridden, debilitated, or suffering from malignancy or undergoing radiation/chemotherapy, subjects unwilling to undergo complete workup or provide written consent, subjects with active infective diseases such as tuberculosis or herpes zoster, and subjects exhibiting evidence of active infection in the concerned eye or the contralateral eye.

### 2.3. Bowman Layer (Membrane) Harvesting

Bowman’s membrane was harvested from therapeutic-grade donor corneas obtained from a certified eye bank. Donor corneas selected for this procedure were from individuals over 40 years of age, as the Bowman layer tends to increase in strength and thickness with age, making it more suitable for surgical applications. The corneal button was mounted on an artificial anterior chamber (Katena, Parsippany, NJ, USA), which was then filled with air to maintain adequate intra-chamber pressure. Following this, the corneal epithelium was carefully removed to expose the underlying Bowman layer. Trypan blue dye was applied to enhance visualization of the membrane. Stromal emphysema was induced to help delineate the Bowman layer from the underlying stroma. A superficial circular marking, typically 9.5 mm in diameter, was created using an appropriately sized trephine. A small flap of the Bowman membrane was then elevated using a crescent knife, and blunt dissection was carried out using an iris repositor. Once sufficient separation was achieved, the remaining membrane was gently peeled away using curved forceps.

### 2.4. Surgical Technique

**Figure 1:**
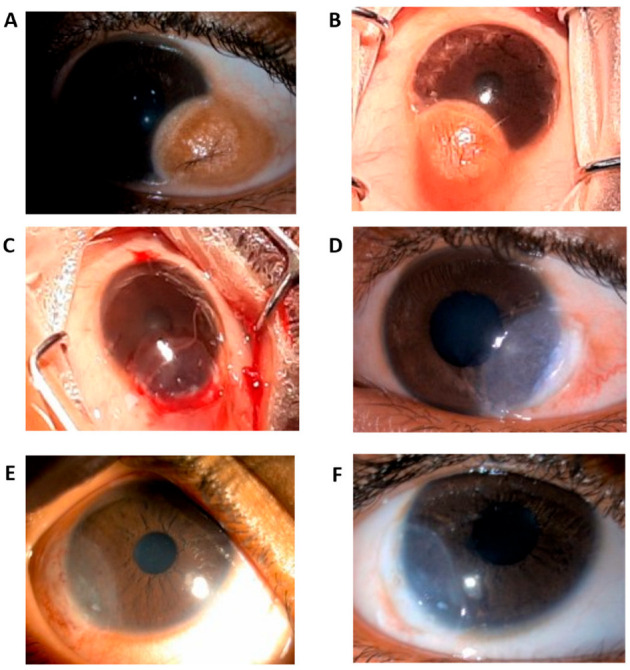
Photograph illustrating the excised limbal dermoid and the surgical steps for its removal and reconstruction. (**A**,**B**) Gross images of two limbal dermoid cases with multiple visible hairs, measuring 4 × 5 mm. (**C**) Complete excision of the dermoid, Bowman’s membrane lenticule filling, and limbal reconstruction with bandage Contact lens. (**D**) Day 1 post-operative with visible limbal stem cell palisades. (**E**) Postoperative external photograph taken four weeks after surgery, showing improved corneal clarity and epithelial healing. (**F**) Postoperative external photograph taken three months after surgery, demonstrating stable limbal architecture and absence of recurrence.

The surgical excision was performed under aseptic conditions. The dermoid tissue was excised up to the level of near-clear cornea, while fatty tissue infiltration on the scleral side was meticulously removed with the help of a crescent knife (Figure 1). A Bowman’s membrane (BM) lenticule was harvested from a therapeutic grade cornea (donor age more than 40 years) received from a certified eye bank and cut fashioned in shape to match the corneal defect created by excision, with an additional 1 mm margin for secure tucking- in along all edges, including the limbus. Limbal reconstruction was performed by the AutoSLET procedure by transplanting limbal stem cells harvested from the same eye (typically a 1 × 1 mm biopsy size of limbal epithelium) from a different, healthy limbal region, preferably from the same eye. All procedures were performed under general anesthesia for children and local anesthesia for adults.

### 2.5. Postoperative Course

A small cystic space was observed between the corneal bed and BM graft on anterior segment optical coherence tomography (AS-OCT) from postoperative day 2–3, which persisted for approximately 1 to 2 weeks post-operatively. Initial follow-ups showed no significant impact on visual acuity; however, gradual improvement was noted over time.

Postoperative treatment included preservative free antibiotic drop (Moxifloxacin 0.5%) 4 times a day and a lubricant drop 4 times a day both until complete healing.

### 2.6. Statistical Analysis

The compilation of results was performed using a proforma designed within a Microsoft Excel spreadsheet (Microsoft, Redmond, WA, USA). The data entries were meticulously rechecked to minimize human error during typing. Statistical analysis was conducted using Statistics for Windows version 22.0 (SPSS Inc., Chicago, IL, USA). Quantitative data were recorded as mean ± standard deviation. A paired *t*-test was employed to compare preoperative and postoperative quantitative data. A *p*-value of less than 0.005 was considered statistically significant.

## 3. Results

A total of 34 subjects participated in this study, with 24 females (75.59%) and 10 males (29.41%). The age of the subjects ranged from 3 to 22 years, with an average of 8.33 ± 6.47 years, reflecting the pediatric nature of the condition (Table 1).

### 3.1. Epithelial Healing

All subjects in the study achieved complete epithelialization following surgery, with a mean healing time of 3.36 ± 0.74 weeks. Epithelial closure was confirmed by the resolution of a transient interface edema, which appeared as a cystic space on AS-OCT in most cases and typically resolved over 3 to 4 weeks. No subjects developed delayed healing or persistent epithelial defects during the follow-up period. These observations suggest that the applied technique provides a supportive environment for consistent epithelial recovery.

### 3.2. Visual Outcomes

Given that limbal dermoids primarily affect the peripheral cornea, the visual prognosis was encouraging. Before surgery, the mean BCVA was 0.136 ± 0.121 decimal units (Table 2). After the procedure, BCVA improved significantly to 0.336 ± 0.214 (*p* < 0.001). This substantial improvement highlights the potential of this surgical approach in restoring functional vision, especially in cases where the visual axis remains unaffected.

### 3.3. Corneal Thickness and Structural Changes

Corneal thickness showed a measurable increase following reconstruction. After dermoid excision, the mean intraoperative corneal thickness at the site of the defect was 294 ± 49.68 µm. Following placement of the Bowman’s membrane lenticule and healing, the postoperative corneal thickness increased to 484 ± 5.037 µm (*p* < 0.001). While this increase is expected due to graft addition, it reflects the structural support provided by the Bowman’s layer and associated stromal remodeling. Despite some residual peripheral thinning noted on Pentacam imaging, no clinical signs of corneal ectasia or structural instability were observed during the 12-month follow-up period, although quantitative keratometric data were not included in this analysis.

### 3.4. Postoperative Observations and Complications

- Early Postoperative Changes: A small cystic space was detected between the corneal bed and the Bowman’s membrane graft on AS-OCT around postoperative day 2–3. This cyst persisted for about one to two weeks before resolving naturally without intervention.
- No Major Complications: Importantly, no cases of graft rejection, infection, or persistent inflammation were observed throughout the follow-up period.
- No Need for Additional Surgery: All subjects responded well to the procedure, and none required further surgical intervention during the study duration.

### 3.5. Overall Implications & Novelty

These results demonstrate that limbal dermoid excision with Bowman’s membrane (lenticule) transplantation is a promising technique, offering rapid healing, significant visual improvement, cosmetic appearance and structural reinforcement of the cornea. While mild peripheral thinning was noted, the long-term stability and functional outcomes remain highly favorable. Limbal stem cell area disruption due to dermoid per se may be corrected by adding AutoSLET procedure for reinstating sectoral limbal niche and stem cells over the Bowman lenticule.

## 4. Discussion

Various surgical techniques have been described for the removal of limbal dermoids, including bare sclera excision, excision with amniotic membrane transplantation [1], and excision with corneal grafting [6]. The traditional surgical approach involving simple excision, while technically straightforward, has been associated with complications such as persistent epithelial defects, localized ectasia, corneal vascularization, and scar formation, which are now attributed to focal marginal limbal stem cell deficiency at the site of dermoid excision [7].

Lamellar keratoplasty, commonly indicated for deep-seated dermoids, has been associated with mild graft opacification, pseudopterygium formation, persistent epithelial defects, and peripheral corneal vascularization [9]. Similarly, procedures involving lamellar keratoscleroplasty with full-thickness central corneal grafts have reported complications such as prolonged re-epithelialization, interface neovascularization, graft rejection, and steroid-induced glaucoma [10]. Another approach, using lamellar dissection followed by reconstruction with fibrin-amniotic membrane, has demonstrated beneficial outcomes [9,11]. However, the inherent short lifespan of amniotic membrane (dissolving within two weeks) often leads to corneal ectasia, surface irregularity, fibrovascularization, and abnormal epithelialization, limiting its long-term efficacy.

### 4.1. Bowman’s Membrane as an Alternative Graft Material

In our study, Bowman’s membrane (BM) transplantation following dermoid excision showed promising anatomical and functional outcomes. BM, being a strong and natural corneal layer, served as a biological patch capable of covering defects of varying size, shape, and thickness [12]. A major challenge following dermoid excision is the tendency of conjunctival cells to proliferate over the raw surface, leading to fibrovascular membrane formation, persistent inflammation, and conjunctivalization. BM together with Auto-SLET effectively mitigated this by facilitating the migration, propagation, and stabilization of epithelial cells, thereby promoting early wound healing, inhibiting vascularization, and preventing abnormal fibrovascular overgrowth [8].

Another advantage of BM is its acellular nature, which minimizes the risk of immune rejection and foreign body reactions. Additionally, limbal epithelial stem cell transplantation using the AutoSLET technique played a crucial role in restoring a healthy limbus and functional epithelium. By directly delivering active limbal stem cells onto the newly applied BM, epithelialization was consistently achieved across all subjects. The average time to complete surface healing was 3.36 ± 0.74 weeks. While this healing duration may be longer than the epithelialization typically reported with procedures using amniotic membrane (which may dissolve and allow re-epithelialization in 1–2 weeks), it reflects the biological integration process of the Bowman’s membrane and the gradual migration and stabilization of limbal stem cells. Compared to techniques involving bare sclera or amniotic membrane alone, the combined use of BM and AutoSLET may offer more robust long-term epithelial integrity and resistance to conjunctival overgrowth, at the cost of a slightly prolonged initial healing period. These results align with recent studies on Bowman’s membrane use in other corneal pathologies, where healing is gradual but structurally favorable [8,13,14]. Our study extends these findings to limbal dermoid excision, demonstrating that BM serves as an effective biological scaffold that not only prevents conjunctival overgrowth and fibrovascular tissue formation but also supports limbal stem cell migration and epithelial stabilization.

Previous research has shown that autologous biological treatments, including buffy coat injections, can accelerate corneal healing by providing growth factors and immune-regulatory components [15]. The combination of BM and AutoSLET thus represents a regenerative approach, leveraging natural corneal components to restore both function and structure [16]. The increase in corneal thickness observed postoperatively is an expected outcome following any form of graft reconstruction, whether using Bowman’s membrane, amniotic membrane, or conjunctival tissue. However, the consistent postoperative thickness values, along with the absence of irregular astigmatism or corneal instability on imaging, suggest that the BM lenticule not only restores volume but also contributes to biomechanical stability. While amniotic membrane is more commonly used due to its availability and anti-inflammatory properties, its short in vivo lifespan (typically dissolving within 10–14 days) may limit its structural contribution, especially in pediatric eyes. Bowman’s membrane, by contrast, provides a longer-lasting stromal scaffold with stronger biomechanical support. However, comparative clinical trials and longitudinal follow-up are necessary to determine whether this structural reinforcement is maintained over time, especially in pediatric subjects.

### 4.2. Postoperative Observations and Study Limitations

One interesting postoperative finding in our study was the appearance of a small cystic space between the corneal bed and BM, as observed on anterior segment optical coherence tomography (AS-OCT). This cyst typically appeared within the first 2–3 days postoperatively and persisted for approximately two weeks before resolving spontaneously. The underlying mechanism of this transient cyst formation remains unclear, but its resolution without intervention suggests a self-limiting healing response rather than a pathological complication.

The small sample size and inclusion of only Grade 1 dermoids limits the generalizability of our findings. Additionally, the lack of pre- and postoperative keratometry and refractive data precludes conclusions regarding the effect on astigmatism. Objective confirmation of limbal stem cell function using modalities such as in vivo confocal microscopy or impression cytology was not performed and represents an important direction for future research. Finally, while we present Bowman’s membrane as a potential alternative scaffold, it should be emphasized that lamellar patch grafts derived from donor cornea, already widely used, also show excellent outcomes in the literature.

## 5. Conclusions

Our findings suggest that Bowman’s membrane tuck-in combined with limbal stem cell reconstruction may be a viable option for corneal surface reconstruction in selected Grade 1 limbal dermoid cases. While preliminary outcomes are promising, especially in terms of epithelialization and structural support, these results must be interpreted with caution due to the small sample size and lack of long-term refractive and topographic data. Further studies involving larger cohorts, multiple dermoid grades, and comparative evaluation with established techniques such as lamellar patch grafts and amniotic membrane transplantation are necessary to better define the role of this technique in clinical practice.

## Figures and Tables

**Table 1:** Demographic Data and Clinical Characteristics.

Parameter	Value
Age (years)	8.33 ± 6.47
Sex	Male (10, 29.41%)/Female (24, 75.59%)
Epithelization Time (weeks)	3.36 ± 0.74
Ulcer Size (mm)	4.1 × 4.0 ± 0.20
Comorbidity	Goldenhar syndrome (1, 2.94%)

**Table 2:** Surgical Outcome Measures: Preoperative vs. Postoperative.

Parameter	Preoperative Value (Mean ± SD)	Postoperative Value (Mean ± SD)	*p*-Value
Best Corrected Visual Acuity (BCVA)	0.136 ± 0.121	0.336 ± 0.214	<0.001
Corneal Thickness (µm)	294 ± 49.68	484 ± 5.037	<0.001

## Data Availability

The data generated and analyzed during the current study are available from the corresponding author upon reasonable request.
